# An Examination of the Medical Regulations Commonly Called the Medical Boon, &c. &c.

**Published:** 1843-10-01

**Authors:** 


### AN EXAMINATION OF THE MEDICAL REGULATIONS COMMONLY CALLED THE MEDICAL BOON, &c. &c. dated July 16th, 1842. By MADRAS MEDICAL OFFICERS. London, 1843.

MANY a good cause has been sadly damaged by injudicious advocacy. It is a recognised rule with all right-minded and prudent men, when they would make an impression upon their superiors, to eschew the slightest approach to disrespect. The authors of this silly pamphlet would seem to be guided by a very different principle. They rely, not upon strong facts, but strong language, to carry the points at issue between themselves, and their honourable masters. The foolish instinct of insubordination and disrespect is so strong in them that it breaks out even in the motto upon their title-page, by which they would insinuate that the masters whose salt they have eaten, and are still eating, for aught we know to the contrary, are upon a par of honesty with the highland freebooter, Rob Roy. The next time they are inclined to discuss matters that have at least a claim to be considered in a temperate and gentlemanly spirit, we would recommend to their attention for a motto a distich quite as suitable and a little older than the verse of the venerable Wordsworth—

> "Ah wud some power the giftie gie us
> To see oursels as others see us!"

The production we are now very reluctantly constrained to notice, has originated in a sentiment of alarm among those whom it chiefly concerns, lest the Marquis of Tweeddale should fill up vacancies in the grade of superintending surgeons, not after "the good old" seniority plan, but upon the newly-acknowledged principle of qualification for the office. Does not this look awfully like a vivid consciousness of demerit upon the part of the Medical Repealers? Why otherwise should the very idea of merit, and not mere length of service, excite such apprehensions, unless there was a consciousness of their liability to be brought to a balance in which they might be found wanting? Admitting that the authors of this pamphlet have some just cause of complaint, let us see how they acquit themselves. They thus introduce themselves to our attention:—

"A NUMBER of Madras Medical Officers having examined, with much care, the new Medical Regulations commonly called the 'Medical Boon'—are of opinion—1st. That the enactments of these regulations are at variance with those principles of strict honour, and integrity of purpose, which hitherto have always formed a distinguishing feature of that Court's orders, on all questions relating to the army; and, 2ndly. That they are so defective in those attributes of wisdom and correct judgment which have ever characterised its awards in all matters of the kind submitted for its decision, that we are forced, almost in spite of our conviction to the contrary, to adopt the opinion, that absolute purity of motive did not actuate the framers, nor preside at the council table at which these orders were passed."

Further on, we have these *officers* thus decorously expressing themselves:—

"That order, as emanating from a body of sensible, well-informed, and educated men, we unhesitatingly pronounce alike digraceful to them, and humiliating to us as professional men and gentlemen."

If the bitterest enemy of the medical service could wish to injure it in the most serious manner, he could not have hit on a more ingenious mode of doing so, than by exciting foolish members of it to write in this fashion respecting grievances real or supposed. It is with feelings of unfeigned pain and regret that we read the passages cited. They might be palliated in an ephemeral print upon the spot, as a passing expression of excited feeling on the part of some individual, " a triton of the minnows," vain of his supposed capacity to produce a storm in a puddle:—viewed, however, as a commodity for exportation, and as a deliberate attempt to set the Thames on fire (the pamphlet is printed in London!), we do not know which predominates, our amazement at the vulgar folly of the perpetrators, or our disgust at their exceeding bad taste. To suppose that such sentiments or language as we have given specimens of, could, by any possibility emanate from the Madras Medical Service, would be a libel upon a highly honourable body. They, when called upon to comment upon any measures of the Government, would be scrupulously guarded to express themselves in language becoming officers and gentlemen. The production before us, then, must by no means be considered as representing the sentiments of the *M*adras Medical Service, but as the ebullition of a small clique of malcontents, dissatisfied, and not without very cogent reasons, we dare be sworn, with the new order of things, which interferes sadly with " the good old rule, the *simple* plan," (exceedingly simple!) of allowing worthless blockheads to lord it, administratively, as chiefs in military circles of superintendence, or divisions of the army; without any reference to qualifications, or rather the *dis*qualifications, they might possess for the important duties of the office.

The pamphlet is not only written in a bad and invidious spirit, but it is ill-timed, to say nothing of its being fraught with ingratitude.* It comes immediately upon the heels, as it were, of a solid and real boon, let these malcontents say what they will, and is thus calculated, not only to make the Court of Directors and Board of Controul regret that they took so much trouble to do a thankless thing, but to make them determine that this should be their final measure of amelioration, and that, however inclined previously to modify what might be pointed out to their satisfaction to be an omission or defect, they would take their stand upon it, defects and all. We say it is calculated to produce such a result, but we trust they know their medical servants better than to suppose that they could, as a body, be cognisant of such an insult to authority, much less sanction it with their approval. As the French politician said, the pamphlet is worse than a crime, it is a mistake—for much that is true in it, (and others have urged these truths over and over again,) is in such close juxta-position

---

* The Boon obtained for the Indian Medical establishments, by Mr. Martin, confers on Surgeons, who formerly had but a retiring pension of £191. annual pensions of £300.—£365.—£500, and £700. What will our English readers think of the Indian manner of receiving so considerate and munificent a grant!

with what is puerile, vulgar, and insolent, as to render the first utterly ineffective—and so we dismiss it.

The Medical Establishments of the three Indian Presidencies undoubtedly laboured under several disadvantages (and partly do so still), partly caused by the force of changing circumstances, partly by the clogging tendencies of time, partly by the naturally slow movements of reform, and partly by misconceptions on some points in high quarters. Various memorials from time to time were submitted on the subject. There is, it is to be feared, a natural reluctance in the human mind to entertain complaint even when it is just. When much press of other business prevails, this inertness to the consideration of complaint, especially when pertinaciously reiterated, will rather grow than diminish. If this be the case with individuals, it is still more so with collective bodies. He who is well acquainted with the machinery of the India Boards can readily understand how a memorial may slumber in their bureaus; this may be more emphatically said of *medical* memorials in departments where a vast aggregation of subjects considered of much higher interest may be pressing for decision, add to which that such memorials rarely if ever receive a cordial, thorough, and earnest momentum from the local governments. This we say advisedly, and more in sorrow than in anger, for the medical service owes but little to the personal good offices of Governors-General or their Secretaries. We say it then, but not at all in a querulous spirit, that *all* medical memorials and all that related to the medical class and its bearings, and reactive results on military combinations, have hitherto been deemed of far more secondary importance than they deserved. Indeed it had been well for the service and well for the state if an individual, who had attained experience in India in the medical service, and who commanded the respect, not only of his own corps, but of his fellow servants in the civil and military departments, and possessing a sufficient knowledge of business as well as tact, temper and judgment, had been always attached to the India House as medical secretary. The appointment of such a functionary would be some security to the service that their interests and claims would not be unsympathisingly overlooked, or coldly slurred over with an official put off on the part of a purely civil or military secretary. All claims in every public department whatsoever require a certain degree of personal influence and energy to impel them through that stagnation that so easily besets them. Fortunately for the Indian Medical Services, though "neither at home nor abroad are their interests represented in the councils of the state," their claims at length found a more efficient fulcrum and lever to move them than their mere abstract value in the opinion of men in office. An individual of their own body but possessing that independent status, morally and socially, without which no man will be listened to in high quarters, be his abilities what they may, generously stepped forward to advocate the cause of his brethren before the India Board. Mr. J. R. Martin has fared like too many reformers. He has not reaped as he sowed, and if there be many (which we cannot for the honor of the Services believe) of the Madras pamphleteers' opinion, then is Mr. Martin likely to be less thanked than blamed for his laborious, unrelaxing, and successful efforts in procuring the Medical Boon for a body to which he no longer belongs. But for these efforts, we can inform our readers as a fact they need have no doubt of, the Boon would have been still a thing not that is, but that might perchance *be* at some future epoch. Let any man but just try to move bodies like the India Boards, and he will then, but not till then, be qualified to understand the amount of personal trouble Mr. Martin went through in effecting an object from which he has derived not the slightest personal benefit, though he has been the active, judicious, and able instrument of conferring much upon a whole service of which he was so truly distinguished a member.* No candid member of that service can deny that it required a renovation of spirit and an amelioration of tone. Mr. Martin's views as to what concerned the real respectability and honor of the service were as wide as the poles asunder from those propounded by such writers as we have alluded to. In his statement of the general subject submitted to the India Chairs, he clearly set forth the condition of the medical service in the following terms.

"While the organization of the Indian Army has been variously and advantageously modified of late years, upon these improvements, the suggestions of experience which have been adopted in the Royal Service, that of the Medical Department remains stationary; and up to this hour it is deemed to have no claim to any other mark of distinction than is included in the mere routine of promotion by seniority:—in fact, the stagnation of this anomalous principle weighs upon it with a peculiar and deadening effect—especially in Bengal, where promotion is so far more slow than at the other Presidencies. The great body of the service—debarred the legitimate expectation and hope of rise or distinction, beyond such as belongs to a name borne so many years on the muster-roll—rather than to a character for knowledge, activity, and discernment—droops and stagnates—so as, after a time, to lose that salutary zeal which prompts to those exertions that, in all other services, secure a just and honourable reward.

"Disadvantages press on every grade of the service; they comprise exclusion from the honors and privileges attached to military advancement, though medical officers be equally exposed with their military brethren to the most pressing dangers of Indian service, as evinced by the numbers

---

* "When Mr. Martin presented himself at the India House in support of the extension to his brother officers of the pension increasing according to length of service, and of the abolition of seniority as a ground for staff promotion—a ground as unwise and injurious in itself as opposed to the standing orders of the Bengal Army—he was received with the utmost attention and consideration by every member of the Direction. His demands were just, and they were readily conceded.

"It was another affair to obtain even a hearing from the Indian Minister of the Crown.

"Here he had to contend with difficulties and delays of a nature impossible here to relate, for a whole year; and when these had all been overcome, and the Minister satisfied and ready to act, a change of parties in the State took place, whence another year, more delays and difficulties, and more explanations were required. The new Minister, however, like the former, satisfied himself of the entire justice of the demands made upon him; but it is not too much to say that, but for the laborious and persevering exertions of Mr. Martin, no boon whatever would have been obtained for many a long year to come."

that fell in the Burmese, Affghan, and Chinese campaigns, as well as in the earlier campaigns of Lord Cornwallis—a low military rank—a too low rate of retiring pension, as well as the want of a greater number of grades; in short, the entire Service demands a re-modelling, to keep pace with the general improvement of the Indian Army, and to assimilate it to the medical service of Her Majesty's Army."

Mr. Martin testifies to the value of the seniority principle in a passage which forcibly illustrates its working, and the allusions of which we know, not from vague hearsay, but from personal experience, to be founded on facts notorious to all who are acquainted with the history of the service (at least at one Presidency) for the last thirty years.

"Promotion to offices of trust should no longer be granted on the seniority principle. To demonstrate the truth of this cannot be necessary, after what has been already stated; but I may as well mention that, in Bengal, an individual of little worth, whether regarded as a medical officer or a gentleman, may now look upon it as certain that, provided he survive his contemporaries, he shall rise in due time, not only to the superior staff employs, but to the governing head of the service; and thus have risen within the writer's recollection, not once, but frequently, the mere merchant, the confirmed gambler, and the exhausted tippler."

"The depressing influence of such a system, is not to be told; and it ought to be henceforth a rule of the Service that no officer, but one of the highest character, moral and professional, should ever rise to such offices: and experience, with approved service with the troops, both in the field and in cantonment, ought to be a qualification in no instance to be dispensed with."

On the appointment to his high office of the Director-General of the Army, we learn* that he received the following instructions from the Commander-in-Chief.

"All promotions and appointments in the medical department of the army to be recommended by you to the Commander-in-Chief."

"You will in your recommendations for promotion be governed by the usual rule of seniority, *as far as in your judgment* circumstances will permit.

"You will *select* and submit for the Commander-in-Chief's approbation all officers and persons to be employed in the medical department abroad and at home."

Let us hear what the same eminent person says in regard to his principle of selection to the higher offices of the Army Medical Department, "the course of promotion in which is regulated throughout upon a principle of selection."

"Unless by the proceedings of a Court of Inquiry or of a Court Martial, there appears anything to warrant an officer being passed over, I invariably adopt the rule of seniority in the service, in recommending the promotion of assistant-surgeons to regimental surgencies, of regimental surgeons to staff surgeons, and of staff surgeons to be assistant inspectors of hospitals; *but in recommending to the two higher ranks*, viz. those of deputy inspector-general and inspector-gene-

---

* Report of the Commissioners for enquiring into Naval and Military Promotion and Retirement.

ral of hospitals, *I find it expedient to adopt a different line. As the officers of those two classes are for general and extensive superintendence* on foreign stations, *I think it right to make a selection,* and *to recommend* (from the knowledge I possess of the merits and talents of all) *an officer who unites, with a thorough knowledge of the service, and of the professional duties, talent for arrangement, and habits of business, together with discretion, discernment, and conciliatory manners, and who can from his character in the service command the respect of those acting under him.*"

These are precisely the principles upon which Mr. Martin and all enlightened well-wishers of the Indian Medical Service at large are anxious to see it administered. Let us also turn to the testimony of a Madras medical officer of a *very* different stamp indeed from those we have been reluctantly compelled to quote—one whom it is a privilege and a source of pride to all who have served in India to hail as a *brother officer.* Mr. Annesley states:—"that it is quite impossible the neglected and degraded state of the medical profession in India can have escaped the notice of the most common observer:—that he has long been of opinion that so lamentable a state of things can only have arisen from inefficient administration, caused by a seniority system of promotion—a system, he regrets to say, which has infested the service from the earliest times—even before the institution of Boards in 1786:—that in these times too, a very inferior class of officers were admitted into the service, and gave to it a low tone:—that though there were, at the same time, some individuals of high character and repute, the bad and inferior vastly prevailed:—that, therefore, it is no matter of surprise to find a department that ought, from its paramount importance to the welfare of armies, to stand high in order and estimation, suffered the most serious deterioration:—that, on the other hand, it is matter of wonder how a Government, which could not be ignorant of the defects of the service, should nevertheless, and with such materials, have adopted a system of promotion and administration, for the medical department, at variance with every other department of the service; for instance, the members of council, of military boards, of boards of revenue, and of trade, have ever been selected officers:—that seniority was never thought of but in that department alone wherein it was calculated to do most injury to the public service:—that a system which takes for the control and management of a great department, three men, just as they stand in seniority order—no matter what their character or qualification—cannot be otherwise than bad, as it is quite impossible that any number of men taken consecutively shall possess the professional character, energy, and administrative talent necessary to the welfare of an army:—that officers with all necessary qualifications can however be found, at all times, and in the required numbers, by another system, that of selection:—that public departments always take their tone from their heads, and that the efficiency with which business is carried on depends entirely on these heads:—that if discretion and energy be wanting at the head, the details of every branch must of necessity be badly conducted:—that it is so, and has long been so, in India:—that the Board of Madras was governed by such feelings that no person could have confidence:—that they showed no knowledge of their profession:—that in his time the members themselves were quite aware of all this:—that the entire system adopted by the Board was confessedly bad:—that when the Board did possess power and patronage it was used, never from public considerations, but from private views:—that men of worth and talent were neglected, while the idle, the careless, the most indifferent, and the most ignorant, were protected and promoted:—that, in short, cringing, idleness, neglect, and ignorance had a better chance, under the Board, than talent and industry:—that, through this depraving system, he has known officers become 'old Indians, with all their follies, before they had been two years in India:'—that the chief boast of the members was the number of years they had lived in India:—that so unworthy was the conduct of the Board, in the important matter of patronage, that this power was obliged to be withdrawn by a special and direct order from the Home Government:—that, however respectable in private life some individual members, Boards constituted like those of India, never have, and never can command the confidence of Government, the respect of the services, or of the profession at large:—that in truth they degrade the profession, and injure the Service:—that it is hoped the recent improvements in the pensions and in the mode of promotion, ordered by the Home Government, will prove beneficial, if the latter be rigorously and systematically acted on:—that whatever the Indian medical services may formerly have been, there were amongst them, and there are now in greater numbers, men of as high a character for talent and experience, as in any public service in the world:—that from such men as these the most efficient staff might at all times, and may now, be selected:—that it is only because men of an opposite character have hitherto been placed in the higher stations that the whole establishment has been contemned, underrated, and undervalued."

"That, after a full and careful consideration of the whole subject, Mr. Annesley would recommend that the medical services of India be made immediately to approximate, as nearly as possible, to that of Her Majesty's army."

It is requisite, for a full understanding of the subject, to give here Mr. Martin's closing summary in his statement to the Chairs of what he conceived the Indian Medical Service required.

1st. That pension, according to length of service, be extended to the medical service of India, the same as granted to the officers of the Indian army, and as accorded by the British Government to the medical branch of the Royal Army; also, that the superior medical officers be entitled to their pensions from the date of their respective commissions, in common with the military officers in India, and with the medical officers of Her Majesty's Service.

2nd. That honors and rewards, proportionate to its services and experience in actual war, be awarded to the Indian Medical Department, and such as are granted to the medical branch of the Royal Army.

3rd. That grades similar to those of the Royal Service be established in the Medical Department of India, and that the Governments be no longer limited in their selection for staff and other medical officers to mere seniority; but that, after the prescribed period of seventeen years' service, all officers of character, and who may have served with credit with troops in the field, be eligible to the higher staff and other employments; professional character, and military experience in actual service, being indispensable.

4th. That no medical officer, of whatever rank, be allowed to continue longer than three years at a civil station, or in civil employment of any kind, with the exception of large cities, as already mentioned.

5th. That the very injurious custom hitherto prevalent, of permitting officers to resign the active branch of the service, still holding for life staff and other responsible offices, be discontinued; and that, for the future, all such as desire to abandon the active duties of the profession be struck off the effective list, and transferred to the Invalid Establishment.

Of these proposals only one, the first, has been carried out, and even that not completely. The original Draft of the Boon, as laid down in the document forwarded by the Military Committee of the Court of Directors to the Board of Control, included a pension of £250. a year after 24 years of service, three for furlough included, but the Controlling Board drew a pen through it, *why* is best known to the Board itself. A service of 17 years in a Tropical climate is so long that, with the changed and changing notions and circumstances of the present day, when steam communication affords a speedy transit to their native land, many will prefer to retire at once, and looking to a cheap residence abroad, or some employ in addition to their pension at home, to the risk of staying on for eight years longer before getting the higher rate of pension. Accordingly some of the ablest men in the service have retired and contemplate retiring before that period, of whom Mr. Martin himself is a striking example.

On the alleged indefeasible "right" to be promoted by "the good old rule, the *simple* plan," of the muster-roll, and on the actually indefeasible "right" of the Honorable East India Company to make what rules and regulations they may please for the promotion and administration of their services, we have said nothing—deeming such discussion a waste of time and of words. Whatever "the good old rule, the simple plan" of rising to seniority honours in the Medical Department of the South may have been, we can assure our brethren there that, to the North and East of them, a less absolute rule has long existed, though unhappily, as Mr. Martin states, it has been allowed to remain a dead letter. But here is the rule:—

"The Governor-General in Council deems it proper to declare, with reference to the principle established by the existing regulations of Government on the subject, and of the great importance of the duties to be performed by superintending surgeons, that the succession to such appointments will not depend on seniority alone, but that the selection will be made with reference to established character for distinguished zeal—strict assiduity, and professional ability, due regard however being had to seniority where not opposed by considerations of a still more powerful nature."—*Bengal Medical Code, Chap.* I, *Section* 2, *Paragraph* 2.

